# CRISPR/Cas9-Based Screening of FDA-Approved Drugs for NRF2 Activation: A Novel Approach to Discover Therapeutics for Non-Alcoholic Fatty Liver Disease

**DOI:** 10.3390/antiox12071363

**Published:** 2023-06-29

**Authors:** James Li, Sandra Arest, Bartlomiej Olszowy, John Gordon, Carlos A. Barrero, Oscar Perez-Leal

**Affiliations:** Department of Pharmaceutical Sciences, Moulder Center for Drug Discovery, School of Pharmacy, Temple University, Philadelphia, PA 19140, USA

**Keywords:** NRF2, NAFLD, HMOX1, CRISPR/Cas9, oxidative stress, fatty liver disease

## Abstract

With the rising prevalence of obesity, non-alcoholic fatty liver disease (NAFLD) now affects 20–25% of the global population. NAFLD, a progressive condition associated with oxidative stress, can result in cirrhosis and liver cancer in 10% and 3% of patients suffering NAFLD, respectively. Therapeutic options are currently limited, emphasizing the need for novel treatments. In this study, we examined the potential of activating the transcription factor NRF2, a crucial player in combating oxidative stress, as an innovative approach to treating NAFLD. Utilizing a CRISPR/Cas9-engineered human HEK293T cell line, we were able to monitor the expression of heme oxygenase-1 (HMOX1), an NRF2 target, using a Nanoluc luciferase tag. Our model was validated using a known NRF2 activator, after which we screened 1200 FDA-approved drugs, unearthing six compounds (Disulfiram, Thiostrepton, Auranofin, Thimerosal, Halofantrine, and Vorinostat) that enhanced NRF2 activity and antioxidant response. These compounds demonstrated protective effects against oxidative stress induced by hydrogen peroxide and lipid droplets accumulation in vitro with hepatoma HUH-7 cells. Our study underscores the utility of CRISPR/Cas9 tagging with Nanoluc luciferase in identifying potential NRF2 activators, paving the way for potential NAFLD therapeutics.

## 1. Introduction

Non-alcoholic fatty liver disease (NAFLD) is the most prevalent hepatic pathology globally, impacting approximately a quarter of the global population [1]. This condition is typified by the excessive accumulation of lipids in hepatocytes, in individuals devoid of significant alcohol consumption, viral infections, or administration of steatogenic medication [2,3]. NAFLD represents a progressive spectrum of disease, potentially transitioning from initial fatty liver to non-alcoholic steatohepatitis (NASH), fibrosis, cirrhosis, and, in some cases, hepatocellular carcinoma [4].

Notably, the co-occurrence of obesity dramatically augments the NAFLD risk, evidenced by a substantial disparity in NAFLD prevalence between obese individuals treated with bariatric surgery (>95%) and the general population (25%) [5,6]. The amplification of oxidative stress levels, attributed to obesity-associated comorbidities such as type 2 diabetes and dyslipidemia, potentiates NAFLD susceptibility. Additionally, the Western dietary pattern, typified by high-fat and high-sugar intake, is frequently implicated in precipitating the global obesity crisis and, indirectly, the burgeoning prevalence of NAFLD [7].

While the comprehensive pathophysiology of NAFLD remains elusive, the ‘two-hit hypothesis’ provides a widely endorsed explanation for the disease’s inception and progression [8]. The ‘first hit’ is embodied by insulin resistance [9], superseded by oxidative stress, the ‘second hit’ [10]. More recent propositions, such as the ‘multiple-hit hypothesis’, propose a confluence of insults including inflammation, which synergistically instigate NAFLD [11]. Oxidative stress, a consequence of the perturbed equilibrium between the production of reactive oxygen species (ROS) and the antioxidant defenses, plays a critical role in NAFLD pathogenesis [1].

The transcription factor, nuclear factor erythroid 2-related factor 2 (NRF2), serves as a master regulator of the cellular defense mechanisms against oxidative stress [12,13]. Under normal physiological conditions, the protein Kelch-like ECH-associated protein 1 (KEAP1) binds to NRF2, promoting its degradation. However, under conditions of oxidative stress, NRF2 dissociates from KEAP1 and translocates to the nucleus. Here, it binds to the antioxidant response element (ARE) in the DNA, stimulating the expression of a large array of genes that encode antioxidant proteins, including heme oxygenase-1 (HMOX1) and NADPH Quinone Oxidoreductase 1 (NQO1) [14,15,16].

Considering the substantial implications of NAFLD on morbidity, quality of life, and healthcare burden, the identification of effective therapeutic strategies is of prime significance. Owing to its crucial role in mitigating oxidative stress, NRF2 has been identified as a potential therapeutic target in NAFLD management. Natural inducers of NRF2 such as apigenin [17] and curcumin [18] have demonstrated beneficial effects in animal models of NAFLD, underscoring the therapeutic promise of NRF2 activation [19].

Two primary strategies are typically employed to discover potential NRF2 activators. The first approach investigates compounds that can modify the interaction between KEAP1 and NRF2 [20], while the second employs reporter cell lines engineered with an artificial antioxidant response element (ARE) that drives the expression of luciferase, facilitating the detection of inducers of the antioxidant response [21,22].

In the current study, we have utilized an innovative approach to identify potential activators of NRF2. By leveraging CRISPR/Cas9 genome editing technology [23], we established a cell line capable of detecting endogenous expression of HMOX1, a frequently expressed target of NRF2 activation. This cell line harbors endogenous HMOX1 tagged with Nanoluc luciferase, which facilitates an efficient and more physiological identification of potential NRF2 inducers. To evaluate the suitability of this cell line for high-throughput compound screening campaigns, we conducted an extensive screening of 1200 FDA-approved compounds. Through this screening, we identified six compounds that were positive for NRF2 activation. Further validation confirmed these compounds’ abilities to enhance the antioxidant capacity of treated cells, increase endogenous NRF2 basal levels, and diminish the accumulation of lipid droplets in an in vitro model of liver steatosis. The findings of this study have the potential to significantly contribute to the development of effective therapeutic strategies for managing NAFLD.

## 2. Materials and Methods

### 2.1. Cell Lines

The HEK293T and HCT116 cells were obtained from ATCC (ATCC, Manassas, VA, USA). HCT116 cells with endogenous C-terminal NRF2 tagged with Nanoluc luciferase were generated in-house and have been previously described [23]. HUH-7 cells were procured from Xenotech (Kansas City, KS, USA). Both HEK293T and HUH-7 cells were cultured in Dulbecco’s modified Eagle’s medium (DMEM) containing 10% fetal bovine serum (FBS) and Normocin™ (Invivogen, San Diego, CA, USA) as recommended by the manufacturer to prevent bacteria, mycoplasma, and fungal contamination. HCT116-NRF2-Nanoluc cells were maintained in McCoy’s medium enriched with 10% FBS and Normocin™. All cells were cultivated in a mammalian cell incubator at 37 °C and 5% CO_2_.

### 2.2. HEK293T HMOX1-Nanoluc Cell Line Development by CRISPR/Cas9 Genome Editing

HEK293T cells (4.5 × 10^5^) were transfected using jetPRIME (Polyplus-transfection; Illkirch, Strasbourg, France) according to the manufacturer’s instructions with plasmids created using the FAST-HDR system [23] for CRISPR/Cas9 genome tagging. The FAST-HDR system consists of two custom-made plasmids. One plasmid expresses CRISPR/Cas9 eSpCas9(1.1) and a targeting sgRNA to induce a double strand break at a specific region in the DNA, while the other plasmid is a donor vector containing recombination arms and a protein tag to facilitate the insertion of the protein tag at the C-terminal of a gene of interest. We developed plasmids for targeting the HMOX1 gene (NCBI gene accession #3162) with the FAST-HDR system, as recently described [23]. The HMOX1 CRISPR/Cas9 sgRNA plasmid was generated by inserting the following oligonucleotide (GCGATCCGAGTTCAAATCTCGGTGGAACCTGTAAGGACCCATCGGAGAAGGTTTTAGAGCTAGAAATAGCAAGTTAAAAT, the sgRNA sequence is underlined) into the plasmid with sSpCas9 (1.1) that we recently reported [23] (Addgene #167210) after digestion with BpiI using the NEBuilder^®^ HiFi DNA Assembly Cloning Kit (New England Biolabs, Ipswich, MA, USA) following the manufacturer’s recommendations. The donor vector promoting the insertion of Nanoluc luciferase at the C-terminal region of HMOX1 was constructed as recently described [23], using the FASTHDR-Cterm-NanoLuc-Puromycin vector available at Addgene (Watertown, MA, USA, plasmid #167209) after doble digestion with BamHI and KpnI. This vector enables the insertion of Nanoluc and the expression of a puromycin resistance gene exclusively in cells with the genomic insertion of Nanoluc following the CRISPR/Cas9 double-strand break. The left and right recombination arms’ sequences were inserted using synthetic DNA fragments made by Integrated DNA Technologies (Skokie, IL, USA) and the NEBuilder^®^ HiFi DNA Assembly Cloning Kit, as recently described [23]. The left arm sequence was 5′ GAC GTT GTA AAA CGA CGG CCA GTG GGT ACC CAT CAT CAC CAT GCT TAG CAA ACG TGT GAG TTT GAG AGG AAG ATT TAC AGC TCA GAC CTA ATT GCT GGC AAA GTT TAA GGA GAG GAC AGG GAG CAG GCA GAA GTC TGA AAA CCA CGC CTG GGC CCA AGA ATG TTT TCA CAA TGT GGC CTG GCT GCA CAG GGA AGA ACA GAC AGC TTG AAG AAG TAG TGA GCT GCC CGT CTT TGA AGG TAT TCA AGC AGT GGC TAG AGG GAC ACC TGT CTG TGG TCT TGC AGA ATC CTG GCG TTG GGC AGT GAC TGT ACC ACA GAC CCT GAG GCC GCT CTG CTT TGC TTT CCT ATG ACA TCA GAC ACC CTG ATG CAC GCC CAC CTG TTA ATG ACC TTG CCC CAT TTT CTC TTT CAG ATT CTG CCC CCG TGG AGA CTC CCA GAG GGA AGC CCC CAC TCA ACA CCC GCT CCC AGG CTC CGC TTG GTA CCC AAG GCG GTG GAG AAT TC 3′, and the right arm sequence was 5′ CAA GTC CCT GCG GTG TCT TTG CTT GGA TCC CTC CGA TGG GTC CTT ACA CTC AGC TTT CTG GTG GCG ACA GTT GCT GTA GGG CTT TAT GCC ATG TGA ATG CAG GCA TGC TGG CTC CCA GGG CCA TGA ACT TTG TCC GGT GGA AGG CCT TCT TTC TAG AGA GGG AAT TCT CTT GGC TGG CTT CCT TAC CGT GGG CAC TGA AGG CTT TCA GGG CCT CCA GCC CTC TCA CTG TGT CCC TCT CTC TGG AAA GGA GGA AGG AGC CTA TGG CAT CTT CCC CAA CGA AAA GCA CAT CCA GGC AAT GGC CTA AAC TTC AGA GGG GGC GAA GGG ATC AGC CCT GCC CTT CAG CAT CCT CAG TTC CTG CAG CAG AGC CTG GAA GAC ACC CTA ATG TGG CAG CTG TCT CAA ACC TCC AAA AGC CCT GAG TTT CAA GTA TCC TTG TTG ACA CGG CCA TGA CCA CTT TCC CCG TGG GCC ATG GCG GAT CCC GGG CCC GTC GAC TGC AGA GGC CT 3′. The underlined regions indicate the segments used for guided DNA assembly into the donor plasmid. A total of 1 µg of each plasmid (eSpCas9[1.1] and HDR Vector) was used per well in a six-well plate.

Seventy-two hours post-transfection, cells were harvested and seeded onto a 100 mm dish, followed by stimulation with an NRF2 activator (CDDO-me, 100 nM) to induce HMOX-1 expression, which is typically low. CDDO-me treatment commenced 24 h before initiating the selection of genetically modified cells using 3 µM puromycin. Culture medium was replaced every 48 h. Cells were grown in the presence of puromycin for seven days, with CDDO-me treatment continuing throughout the selection process. After selecting a pool of modified cells, the use of puromycin and CDDO-me was discontinued.

A clonal cell line was derived from the modified pool of cells using single cell sorting with a Namocell Hana Single Cell Dispenser Instrument (Namocell, San Jose, CA, USA), following the method we recently described [24]. Briefly, a cell suspension at a concentration of 5000 cells/mL was employed for single cell sorting into 100 µL of culture media per well in a 96-well plate, utilizing the forward scattering detection mode. Five days post-sorting, an additional 100 µL of media was added to each well, and individual cell clones were identified at day ten by whole well imaging using a Tecan Spark Cyto (Tecan, Männerdorf, Switzerland) multimode reader, as described previously [24].

The insertion of Nanoluc luciferase at the C-terminal end of HMOX1 was confirmed through PCR analysis of individual clones. Culture media from each clone were removed, and cells were treated with 25 µL of Tryple Express (Thermo Fisher Scientific, Waltham, MA, USA) for five minutes. From each well, 20 µL of cell suspension was transferred to a 24-well plate containing 500 µL of culture media per well for clonal expansion. The remaining 5 µL of cell suspension was mixed with 25 µL of QuickExtract DNA extraction buffer (Biosearch Technologies-LGC, Middlesex, UK), followed by heating for six minutes at 65 °C, vortexing, and heating for two minutes at 98 °C for rapid DNA isolation.

Primers were designed to detect HMOX1 tagging using the NCBI primer design tool: https://www.ncbi.nlm.nih.gov/tools/primer-blast/ (accessed on 15 February 2023). Forward (5′ ACC AGG GAT GGG ACT GAA CT 3′) and reverse primers (5′ ACG CAA GAA ACC AGG GAG GA 3′) were created to anneal approximately 500 and 700 bp upstream and downstream, respectively, of the CRISPR/Cas9 targeting site in HMOX1. Additionally, a reverse primer (5′ TCC CAC GAA GTC CTC CAA CGT AAA CAC C 3′) annealing to the Nanoluc gene sequence of the HDR plasmid was included in the polymerase chain reaction (PCR) mixture to produce a PCR product of around 530 bp if the Nanoluc luciferase insertion occurred at the HMOX1 targeted site.

To prepare the PCR master mix, we calculated the amount in microliters per sample of the following components: 0.75 µL of forward primer, 0.625 µL of reverse primer, 0.625 µL of reverse Nanoluc primer, 6.25 µL of Q5^®^ High-Fidelity 2X Master Mix from New England Biolabs, and 3 µL of water. The components were mixed and aliquoted 11.25 µL per sample. Lastly, 1.25 µL of the extracted DNA solution from each clone was added to the corresponding tube containing the master mix. Furthermore, PCR-positive clones were expanded and assessed for Nanoluc luciferase expression with and without an NRF2 activator (CDDO-me, 100 nM) for 24 h.

### 2.3. Chemicals

We used the Prestwick drug library of FDA-approved compounds, consisting of 1200 compounds from Prestwick (Illkirch, Alsace, France), for drug screening. The following compounds of Disulfiram, Auranofin, Thiostrepton, Thimerosal, Halofantrine, Vorinostat, CDDO-me, RTA-408, oleic acid, and palmitic acid were purchased from Cayman Chemicals (Ann Arbor, MI, USA). Hydrogen Peroxide was obtained from MilliporeSigma (Burlington, MA, USA).

### 2.4. High-Throughput Screening and Luminescent Detection Assays

The HEK293T cell line with HMOX1-Nanoluc was used to perform high-throughput drug screening of the Prestwick drug library of FDA-approved compounds. Greiner Bio-One (Monroe, NC, USA) CELLSTAR 384-well white plates were seeded with 5 × 10^3^ cells in 50 µL per well, and 16 h later, the cells were treated with each library compound at a final concentration of 500 nM using a Janus automated liquid transfer workstation (PerkinElmer, Waltham, MA, USA) and incubated at 37 °C and 5% CO_2_. We treated 32 wells per plate only with DMSO as untreated negative controls. The effects of the compounds on HMOX1-Nanoluc expression were evaluated 24 h later using Nano-Glo luciferase (Promega, Madison, WI, USA) as recommended by the manufacturer. The luciferase activity was measured using a Tecan Spark multimode reader (Tecan, Männedorf, Switzerland). Fold changes in HMOX1-Nanoluc expression after treatment with each compound were calculated relative to the average of all the negative control wells in each plate.

To evaluate the efficacy of positive hits in increasing the expression of HMOX1-Nanoluc or NRF2-Nanoluc, a dose–response experiment was conducted using HEK293T HMOX1-Nanoluc and HCT-116 NRF2-Nanoluc cell lines. Fresh powder hit compounds (Disulfiram, Auranofin, Thiostrepton, Thimerosal, Halofantrine, and Vorinostat) were acquired and dissolved in DMSO to evaluate their activity over a concentration range of 1–1000 nM in quadruplicates. Both cell lines were seeded at a concentration of 5000 cells in 50 μL of culture media, followed by the addition of the compounds 16 h after cell seeding to 384-well plates. The cells were then incubated with the compounds for 24 h. Following the incubation period, a luciferase assay was performed using the Nano-Glo luciferase reagent, as recommended by the manufacturer. This allowed for a quantitative assessment of the ability of the compounds to induce the expression of HMOX1-Nanoluc or NRF2-Nanoluc.

### 2.5. Hydrogen Peroxide Cell Viability Assay

To evaluate the ability of identified NRF2 activators to enhance the antioxidant capacity of treated cells, a cell viability assay was performed against treatment with 200 μM hydrogen peroxide (H_2_O_2_) for 3 h. The hepatoma cell line HUH-7 was used for this assay due to its active NRF2 pathway. For the assay, 5000 cells were seeded in 50 μL of culture media in white 384-well plates, followed by the addition of the identified NRF2 activators (Disulfiram, Auranofin, Thiostrepton, Thimerosal, Halofantrine, and Vorinostat) in a two-fold serial dilution starting at 500 nM in quadruplicates, 16 h later. The cells were then incubated with the compounds for 24 h, after which they were treated with 200 μM H_2_O_2_ for 3 h. Cell viability was determined using the CellTiter-Glo assay from Promega as recommended by the manufacturer.

### 2.6. Lipid-Droplet Detection by High-Content Imaging

We evaluated the ability of identified NRF2 inducers to prevent lipid droplet accumulation in HUH-7 cells. We seeded 8000 cells per well in 100 μL of culture media in CellCarrier Ultra 384-well glass bottom plates (Perkin Elmer, Waltham, MA, USA). After 16 h, we treated the cells with each of the compounds (Disulfiram, Thiostrepton, Auranofin, Thimerosal, Halofantrine, and Vorinostat), along with two known NRF2 inducers (CDDO-me and RTA-408), at a uniform concentration of 250 nM for 24 h. This concentration was chosen based on our preliminary findings that demonstrated its consistent effectiveness in activating HMOX1 expression for most of the compounds, and its minimal impact on the viability of HUH-7 cells for most of the compounds. We then stimulated lipid droplet formation by treating the cells with a mixture of oleic acid: palmitic acid (1.5 mM:0.75 mM final concentration) for an additional 24 h as previously reported [25].

Subsequently, the cells were stained to detect the nucleus with Hoechst 33342 (Thermo Fisher) (1 μg/mL), 1X CellMask Deep Red (Thermo Fisher) for plasma membrane detection, and 15 nM BODIPY™ 493/503 (Thermo Fisher) for lipid droplet labeling, followed by incubation at 37 °C for 30 min. The cells were then analyzed using confocal high-content imaging with the Operetta CLS imager (PerkinElmer) at 5% CO_2_ and 37 °C. Images were acquired with a 63X water immersion objective in three independent channels (Hoechst 33342 [Ex-355, Em-440], BODIPY 493/503 [Ex-480, Em-513], and CellMask Deep Red [Ex-642, Em-670]).

The experiment was conducted in triplicates, and nine images were acquired per well for each condition. Image analysis was performed using Harmony 4.8 Software (PerkinElmer) with the following steps: (1) identify nuclei of each cell using the Hoechst 33342 channel, (2) locate the cytoplasm of each cell using the CellMask Deep Red channel, and (3) detect puncta representing lipid droplets in the cytoplasm of every cell by employing the BODIPY 493/503 channel.

### 2.7. Statistical Analysis

All figures presented in this work are representative of at least three independent experiments. To analyze the differences between control and treated samples, a one-way analysis of variance (ANOVA) with Dunnett’s test was performed using GraphPad Prism version 9. A *p*-value < 0.05 was considered statistically significant. The Z-factor [26], employed for determining the quality of the HMOX-1-Nanoluc assay in discovering activators of NRF2, was calculated using readout values for the expression of HMOX1-Nanoluc in cells treated with DMSO (n = 6) or treated with CDDO-me at 500 nM (n = 6). The Z-factor was calculated as follows:

Z factor = 1 − [(3 × SD(CDDO-me) + 3 × SD(DMSO))/(mean(CDDO-me) − mean(DMSO))]

In this formula, SD refers to the standard deviation. This calculation allowed us to assess the suitability of the assay for high-throughput screening applications.

## 3. Results

### 3.1. Development of a Cell Line for Detecting Endogenous Hmox-1 Expression

**Figure 1:**
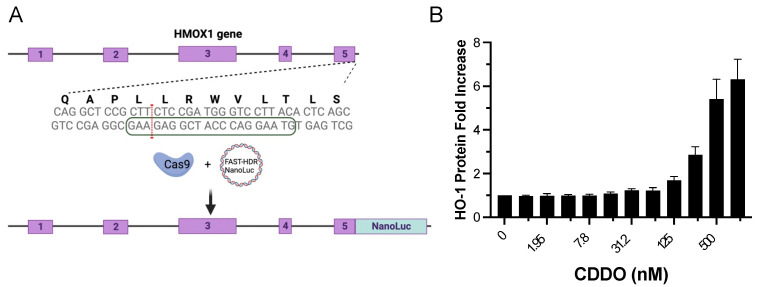
Generation of a CRISPR/Cas9-engineered cell line with endogenous tagging of HMOX1 using Nanoluc luciferase and examination of its response to NRF2 induction. (**A**) Schematic representation of the HMOX1 gene locus targeted for CRISPR/Cas9 induced double-strand break. The exons of the HMOX1 gene are indicated with numbers 1 to 5. The boxed sequence on the lower DNA strand marks the location of the single guide RNA (sgRNA) used to direct CRISPR/Cas9 to the targeted genomic region. The red line signifies the CRISPR/Cas9 double-strand break site, indicating where the insertion of the Nanoluc luciferase tag was inserted. (**B**) Dose–response curve displaying the expression of endogenous HMOX1-Nanoluc luciferase in response to treatment with CDDO-me, a known NRF2 activator. Cells were treated with two-fold dilutions of CDDO-me, starting from 1000 nM, over a 24 h period. The resulting changes in HMOX1 expression were monitored, demonstrating the functionality of the Nanoluc luciferase tag in tracking HMOX1 expression levels.

The identification of NRF2 activators is of great interest due to its crucial role in the cellular resistance to oxidative stress, which has been implicated in the pathogenesis of various diseases, including NAFLD. Here, we developed a new approach to identify NRF2 activators that involves the development of a cell line for detecting endogenous Heme oxygenase-1 (HMOX-1) expression upon NRF2 nuclear translocation. HMOX-1 is a well-known downstream target gene of NRF2 and is the most commonly activated protein upon NRF2 nuclear translocation [22]. To achieve this, we used CRISPR/Cas9 genome tagging with Nanoluc luciferase using the FAST-HDR system [23] to create a HEK293T cell line in which the endogenous HMOX-1 protein is C-term tagged with Nanoluc luciferase (HMOX1-Nanoluc cell line) (Figure 1A). This approach allowed us to detect the endogenous expression of HMOX-1 in response to NRF2 activation by the known NRF2 activator CDDO-me [27], providing a more physiological approach to identify NRF2 activators (Figure 1B). The Z-factor for CDDO-me at 500 nM, which measures the suitability of an assay for high-throughput screening (HTS), was 0.84. Assays with values above 0.5 are considered excellent for HTS [26]. By using this cell line, we aimed to identify NRF2 activators independent of the mechanism that allows those compounds to increase the activity of NRF2, as the activity of NRF2 is regulated by multiple processes including protein degradation [28], translational control [29,30,31,32], and protein phosphorylation [33].

### 3.2. Screening of an FDA-Approved Compound Library to Detect Inducers of HMOX1 Expression

**Figure 2:**
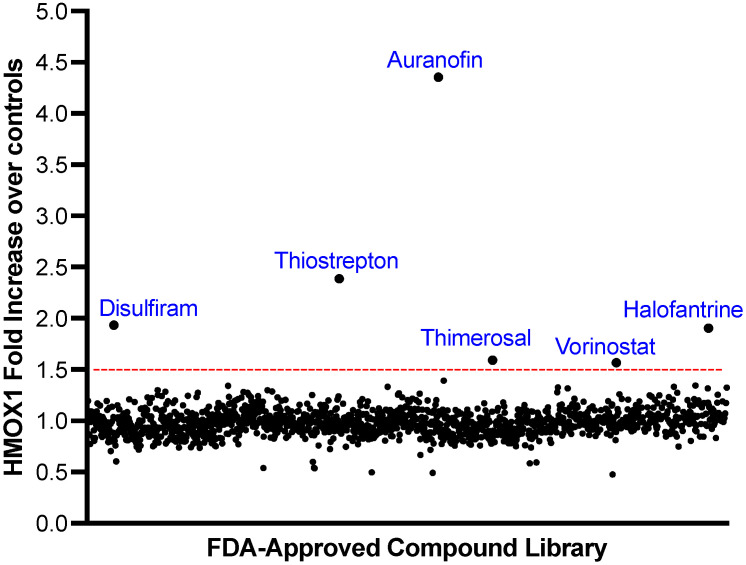
Screening of a Library of FDA-Approved Compounds to Identify HMOX1 Inducers. A library of 1200 FDA-approved compounds was screened at 500 nM over a 24 h period to detect inducers of HMOX1 protein expression. The x-axis represents individual compounds, while the y-axis shows the fold change in HMOX1 expression. A threshold for potential hits, indicated by a red line, was determined as the mean fold change across all compounds plus three times the standard deviation. Out of the entire library, six compounds—Disulfiram, Thiostrepton, Auranofin, Thimerosal, Halofantrine, and Vorinostat—exceeded this threshold.

We aimed to identify potential NRF2 activators from a library of 1200 FDA-approved compounds using a HTS assay with the HMOX1-Nanoluc cell line. For this assay, cells were plated and 16 h later treated with compounds from the library at a concentration of 500 nM for 24 h. Six compounds were identified as potential activators of NRF2: Disulfiram, Thiostrepton, Auranofin, Thimerosal, Halofantrine, and Vorinostat. The fold changes in HMOX1 expression for each compound are displayed in Figure 2.

### 3.3. Validation of Hits That Promote HMOX1 Expression

Following the identification of six potential hits that promote HMOX1 expression, we further validated the capacity of these compounds to activate HMOX1 expression. Fresh powder compounds were obtained for the validation assay, and a dose–response assay was performed for 24 h with each of the compounds in a dose range between 1–1000 nM using the HMOX1-Nanoluc cell line.

**Figure 3:**
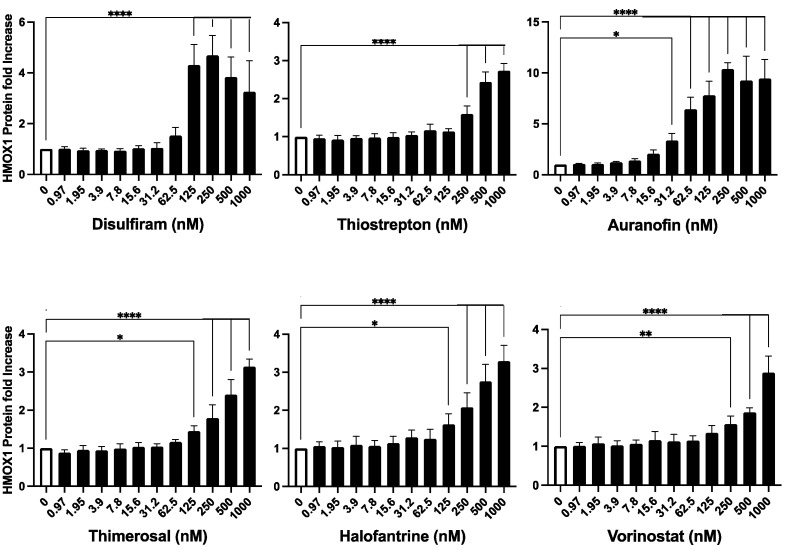
Validation of Identified HMOX1 Inducers. The six compounds identified as potential inducers of HMOX1 expression (Disulfiram, Thiostrepton, Auranofin, Thimerosal, Halofantrine, and Vorinostat) were further assessed in the HEK293T cell line with endogenously tagged HMOX1-Nanoluc. The compounds were applied in a two-fold dilution series starting from 1000 nM and left to interact with the cells for 24 h. The aim was to confirm their capability to induce HMOX1 expression in a dose–response manner. The x-axis of each panel represents the concentration of the compound, and the y-axis denotes the fold change in HMOX1 protein expression. These experiments were performed in quadruplicates and were independently repeated three times to ensure reproducibility. Statistical significance is denoted by: * *p* ≤ 0.05, ** *p* ≤ 0.01, **** *p* ≤ 0.0001.

The results of the validation assay confirmed that all six compounds were able to increase the expression of HMOX1 in a dose-dependent manner. In the tested range, most compounds were able to increase the expression of HMOX1 by up to three-fold at higher doses, with the exception of auranofin, which was able to increase HMOX1 expression up to 10-fold at high doses (Figure 3). Notably, auranofin was able to induce an increase in HMOX1 expression of at least three-fold at low nanomolar doses (15–30 nM).

Overall, our results demonstrate that the six identified compounds, Disulfiram, Auranofin, Thiostrepton, Thimerosal, Halofantrine, and Vorinostat, have the capacity to promote HMOX1 expression in a dose-dependent manner.

### 3.4. Inducers of HMOX1 Expression Increase NRF2 Expression

To examine whether the discovered HMOX1 inducers also enhanced NRF2 levels, we used a recently reported cell line, created using CRISPR/Cas9 genome editing, which has endogenous NRF2 tagged with Nanoluc luciferase [23]. This cell line allows for the detection of changes in NRF2 protein expression. We aimed to determine whether compounds that increase HMOX1 expression do so indirectly by promoting NRF2 expression. For this assay, cells were treated with the compounds for 24 h in a dose range between 1–1000 nM.

**Figure 4:**
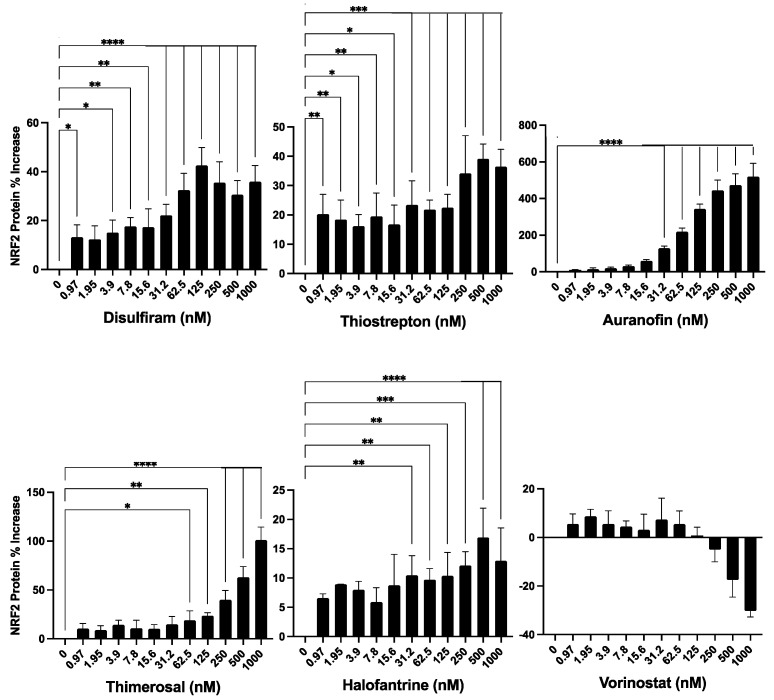
Evaluation of the identified compounds for their ability to induce NRF2 expression. The six compounds previously identified as inducers of HMOX1 expression were further tested for their ability to enhance the expression of NRF2 in a dose-response assay. For this experiment, an HCT -116 cell line with endogenous NRF2 tagged with Nanoluc luciferase was employed, thus facilitating the detection of changes in NRF2 protein abundance. The compounds were administered in a two-fold dilution series beginning from 1000 nM and left to act on the cells for 24 h. The x-axis in each panel denotes the concentration of the respective compound, while the y-axis illustrates the percentage change in NRF2 protein levels from untreated controls after 24 h of treatment. This experiment was executed in quadruplicates and independently replicated three times. Statistical significance is denoted by: * *p* ≤ 0.05, ** *p* ≤ 0.01, *** *p* ≤ 0.001, **** *p* ≤ 0.0001.

Our findings revealed that the compounds induced an increase in NRF2 protein expression, though to varying degrees (Figure 4). Thimerosal and Auranofin led to a significant increase in NRF2 expression, ranging from a 100% to a 500% maximum increase over basal levels. Disulfiram and Thiostrepton, on the other hand, showed a moderate rise in NRF2 expression, with a maximum increase of 20% to 40% over basal levels. Halofantrine and Vorinostat resulted in a low increase in NRF2 expression, with a maximum increase of 5% to 20% over basal levels. It is important to note, however, that the changes in NRF2 expression induced by Vorinostat were not statistically significant. Furthermore, Vorinostat was the only compound that exhibited a suppressive effect on NRF2 expression at concentrations above 125 nM, leading to a decrease in basal NRF2 expression.

### 3.5. Inducers of HMOX1 Expression Protect against Hydrogen Peroxide Oxidative Stress

Inducers of NRF2 have been shown to protect against oxidative stress by enhancing the cellular antioxidant response [34,35,36]. To determine the protective effects of the newly identified compounds against oxidative stress, we used HUH-7 hepatoma cells for evaluating cell survival after a short exposure to a high dose (200 μM) of hydrogen peroxide.

HUH-7 cells were treated with the compounds in a two-fold serial dilution starting at 500 nM for 24 h, followed by treatment with hydrogen peroxide for 3 h to evaluate cell viability. In a parallel experiment, we assessed the effects of each compound on the viability of HUH-7 cells in the absence of hydrogen peroxide.

**Figure 5:**
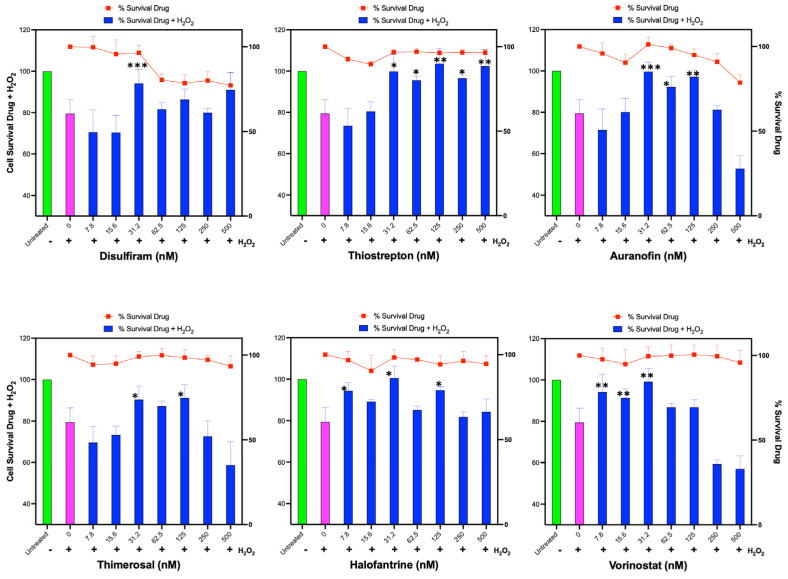
Assessment of Identified Compounds for Protective Effect Against H_2_O_2_-Induced Oxidative Stress. Human hepatoma HUH-7 cells were pre-treated for 24 h with each of the six identified compounds that promote HMOX1 expression (Disulfiram, Thiostrepton, Auranofin, Thimerosal, Halofantrine, and Vorinostat) in a two-fold dilution series beginning from 500 nM. Following this, cells were exposed to 200 µM H_2_O_2_ for 3 h, and cell survival was subsequently measured. Additionally, cell viability was determined in response to the individual drugs alone across the same concentration range after 24 h of treatment, which is represented by the red line in each panel. The x-axis in each panel indicates the drug concentration. The left y-axis is for the bars of the chart and displays the percentage survival of cells post H_2_O_2_ treatment, while the right y-axis represents the percentage survival in response to the drug treatment alone, and it is represented with the red line. These experiments were conducted in sextuplicate and independently replicated three times to ensure the validity of the results. Statistical significance is denoted by: * *p* ≤ 0.05, ** *p* ≤ 0.01, *** *p* ≤ 0.001.

We found that all evaluated compounds ameliorated the reduction in cell viability caused by exogenous hydrogen peroxide, albeit with varying levels of protection (Figure 5). Disulfiram exhibited a protective effect at 31 nM, while Thiostrepton was highly effective in reducing H_2_O_2_-induced cell death between 31 and 500 nM. Auranofin also prevented cell death caused by H_2_O_2_ at doses between 31 and 125 nM. Thimerosal demonstrated a moderate benefit between 31 and 125 nM, Halofantrine was effective in protecting the cells from 7.8 to 125 nM, and Vorinostat was only effective at low doses between 7.8 and 31 nM.

Interestingly, some compounds, such as Thimerosal and Vorinostat, exhibited a detrimental effect on protection against H_2_O_2_ at high doses (250–500 nM) that did not affect cell viability when used alone. Additionally, Auranofin at high doses negatively impacted cells treated with H_2_O_2_; however, this may be related to the toxicity of Auranofin at those doses in the HUH-7 cell line.

These results demonstrate that the identified compounds can improve the antioxidant capacity and cell viability of HUH-7 cells under oxidative stress induced by hydrogen peroxide, although the extent of protection and the optimal dose range vary among the compounds.

### 3.6. Inducers of HMOX1 Prevent Steatosis In Vitro

Oxidative stress has been associated with NAFLD [37,38], and several NRF2 activators have been shown to reduce steatosis in vitro and in vivo [17,18,39,40,41]. To investigate the potential of the identified HMOX1 inducers in this study to ameliorate NAFLD, we set up an in vitro steatosis assay using HUH-7 cells. In this assay, cells are treated with a mix of oleic acid and palmitic acid, which promotes lipid droplet accumulation.

We assessed the protective effect of the identified compounds, along with two potent NRF2 activators as positive controls, in an in vitro steatosis assay using HUH-7 cells. Cells were treated with the compounds at a single dose of 250 nM for 24 h, followed by inducing steatosis with an oleic acid:palmitic acid mix for an additional 24 h. Lipid droplets were stained and analyzed using high-content imaging to determine changes in lipid droplet accumulation in treated cells.

**Figure 6:**
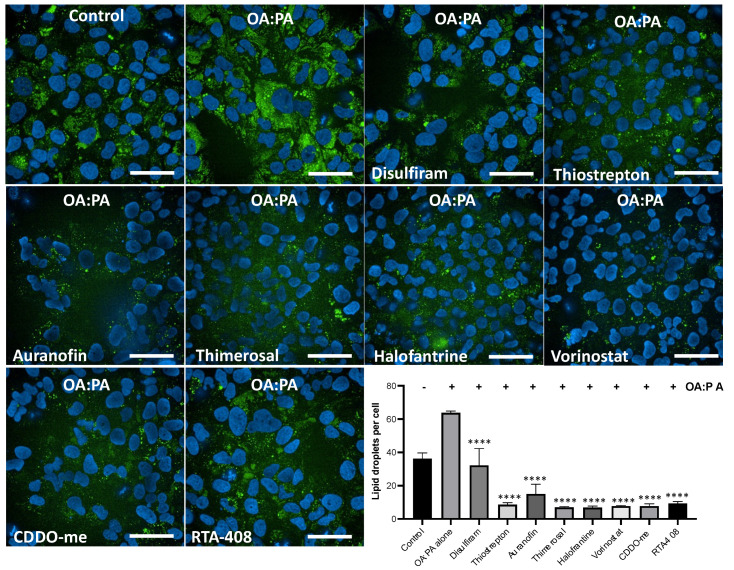
High-content imaging analysis of lipid accumulation reduction by inducers of HMOX1. Using confocal high-content imaging analysis, the ability of six identified HMOX1-inducing compounds (Disulfiram, Thiostrepton, Auranofin, Thimerosal, Halofantrine, and Vorinostat), and two well-known NRF2 inducers (CDDO and RTA-408), to mitigate lipid droplet accumulation in HUH-7 liver hepatoma cells was examined. Cells were initially treated with each compound at 250 nM for 24 h and were subsequently exposed to a combination of lipoic acid and palmitic acid (1.5 mM:0.75 mM) for an additional 24 h to induce in vitro steatosis. Specific cellular segmentation methods and chemical staining were utilized to visualize and quantify lipid droplets. Results from treated samples were compared with those from cells exposed solely to the lipid mixture. Statistical significance, indicated by ****, denotes differences in the number of lipid droplets in treated cells versus untreated controls following exposure to the Oleic acid:Palmitic acid mix (*p* < 0.0001). Experiments were conducted in triplicate and independently replicated three times to validate results. Scale bar = 50 µm.

We found that all the compounds reduced lipid droplet accumulation (Figure 6). Interestingly, all the compounds, with the exception of disulfiram, reduced lipid accumulation as strongly as the known NRF2 inducers. These results indicate that the identified inducers of HMOX1 have the potential to ameliorate steatosis in vitro, further supporting the potential use of NRF2 inducers as therapeutic agents for NAFLD.

## 4. Discussion

In this study, we sought to identify novel NRF2 activators with potential therapeutic implications for NAFLD and other oxidative stress-related diseases. Our approach involved the development of a novel CRISPR/Cas9-engineered HMOX1-Nanoluc cell line, screening of an FDA-approved compound library, and assessment of the identified compounds for their protective effects against oxidative stress and steatosis in vitro. The results of our study have led to the identification of six compounds that induce HMOX1 expression and exhibit protective effects against oxidative stress and steatosis in vitro, highlighting their potential as therapeutic agents for NAFLD.

Our findings are in line with previous research that has implicated NRF2 activation in the amelioration of oxidative stress and NAFLD. Several known NRF2 activators, such as sulforaphane and curcumin, have been reported to reduce steatosis in vivo [18,42]. Interestingly, some of the compounds identified in our study, including Disulfiram [43,44], Auranofin [45], Thimerosal [46], and Vorinostat [47], have been previously reported as molecules that can promote an increase in the levels of NRF2.

For instance, Ren et al. [43] demonstrated that Disulfiram promotes an increase in the phosphorylation of P62 (SQSTM1), and it is known that increasing P62 phosphorylation disrupts the KEAP1-NRF2 interaction [48], thus preventing the excessive degradation of NRF2. In the case of Auranofin, it has been shown to promote an increase in NRF2 in a human monocyte cell line through multiple mechanisms. Primarily, Auranofin disrupts the interaction between KEAP1 and NRF2, thereby stabilizing NRF2 and preventing its degradation [45]. Additionally, Auranofin increases the expression of inducible nitric oxide synthase (iNOS) and enhances the phosphorylation of mitogen-activated protein kinases (MAPKs). Interestingly, the inhibition of iNOS or the inhibition of MAPKs diminished the increase in NRF2 promoted by Auranofin in those cells [45].

Notably, the identified compounds in our study, particularly Auranofin, demonstrated strong induction of HMOX1 expression and protective effects against oxidative stress and steatosis in vitro. Furthermore, recent work has shown that Auranofin reduced liver steatosis and fibrosis in an animal model of NAFLD by activating NRF2 [49]. This evidence suggests that the development of novel NRF2 activators has significant therapeutic potential for NAFLD and other oxidative stress-related diseases.

Our study has not only identified NRF2 activators but also revealed potential candidates for drug repurposing in the context of NAFLD. Most of the identified compounds are FDA-approved drugs with known safety profiles and oral availability. Disulfiram, Vorinostat, and Auranofin, in particular, should be further explored for their potential use against NAFLD due to in vivo evidence in animal models. Disulfiram has recently been shown to improve NAFLD in mice [50]; however, its effect on NRF2 or the antioxidant response has not been explored. Vorinostat, also known as SAHA, was previously identified as a drug that reduces insulin resistance and liver steatosis [51]. Our data indicate that Auranofin demonstrated a clear dose-dependent induction of both HMOX1 and NRF2. Additionally, it was active at low nanomolar levels and was recently reported as effective against NAFLD in mice by activating the NRF2 pathway and reducing the inflammatory response [49]. This evidence suggests that Auranofin should be considered for further testing in humans for treating NAFLD. Given the urgent need for effective treatments for this prevalent and progressive disease, drug repurposing of these compounds could streamline the development process, potentially resulting in a more rapid translation into clinical practice. 

Our study has some limitations, such as the use of in vitro models and the lack of characterization of other antioxidant protein targets of NRF2. Further research into the specific mechanisms of action and effects of these compounds on NRF2 activation and downstream pathways, as well as validation in in vivo studies and pharmacokinetic evaluations, will be essential to fully explore their therapeutic potential for NAFLD and other oxidative stress-related diseases.

## 5. Conclusions

Our study has demonstrated the advantages of using a CRISPR/Cas9-engineered cell line with endogenous HMOX1 with Nanoluc luciferase to discover activators of the NRF2 pathway. The use of endogenous HMOX1 as a readout in our novel cell line provides a more physiologically relevant approach for identifying NRF2 activators, which may lead to the discovery of compounds with better efficacy and safety profiles. This technology can be used to screen large and diverse compound libraries to identify non-toxic activators of NRF2 for their potential use in treating NAFLD and other diseases affected by oxidative stress.

## 6. Patents

The US Patent # 10,883,120 contains partial information from this work.

## Data Availability

The data presented in this study are available upon reasonable request from the corresponding author.
